# Re-enchanting Consumer Ethics Through Embodied Relationality: An Ethnographic Approach to the Attitude-Behaviour Gap

**DOI:** 10.1007/s10551-025-06227-y

**Published:** 2025-12-16

**Authors:** Roberta Discetti, Barbara Tocco, Matthew Gorton

**Affiliations:** 1 Institute for Sustainability, University of Surrey, Guildford, UK; 2 National Innovation Centre for Rural Enterprise, Newcastle University, Newcastle Upon Tyne, UK; 3 Newcastle University Business School and National Innovation Centre for Rural Enterprise, Newcastle University, Newcastle Upon Tyne, UK; 4 Corvinus University, Budapest, Hungary

**Keywords:** Food practices, Embodiment, Family consumption

## Abstract

Most research on the ‘attitude-behaviour gap’ adopts linear, rational, and individual models, or highlights notions of consumer uncertainty and confusion. We argue that different strands of the debate can benefit from an exploration of *embodied relational* processes shaping consumer ethics, namely an acknowledgement of the profound corporeal and interdependent nature of all consumption experiences. The aim of this paper is thus to explore the embodied relational aspects of everyday ethical consumption and cast new light on the ‘attitude-behaviour gap’. We do so by drawing on embodiment in practice theories and through an ethnographic study with six families in North-East England, including observations, interviews, kitchen and walking-with tours, and participants’ diaries. Our findings highlight four embodied relational practices: *reconciling competing embodied states; negotiating togetherness; seeking sensorial familiarity; and calibrating attention and engagement*. The paper contributes to the existing literature by offering *embodied relationality* as a theoretical framework to visualise the complex ambivalent embodied state consumers experience in everyday practices*.* We leverage this to rethink the ‘attitude-behaviour gap’ as a *disenchanted concept*, namely detached from the embodied nature of all social action and, as such, limited in its explanatory potential. We conclude the paper by outlining future research and intervention avenues to *re-enchant* consumer ethics scholarship, namely reconnect it with its embodied and relational nature.

## Introduction

Ethical consumption debates have long been interested in the perceived discrepancy between consumers’ attitudes and actions—the ‘attitude-behaviour gap’. This refers to the inconsistency between what individuals say regarding their ethical concerns, and how they act upon these concerns in their consumption choices (ElHaffar et al., 2020). In this study, we investigate this phenomenon from an embodied perspective, using the body as a methodological and conceptual tool (Wallenborn & Wilhite, 2014).

Most research on the attitude-behaviour gap seeks to identify the factors that determine it using linear, rational, and individual models, most notably the Theory of Planned Behaviour (Ajzen, 1991), as documented in several reviews of the literature (Andorfer & Liebe, 2012; ElHaffar et al., 2020; Fischer et al., 2021; Hassan et al., 2016). However, not all agree with this approach—a complementary body of research argues against individual-level models and reframes the attitude-behaviour gap through the adoption of a more nuanced approach to consumer ethics. The latter highlights that consumers’ everyday lives are ridden with competing ethical priorities, multiple caring commitments, and uncertainty (Carey et al., 2008; Carrington et al., 2014; Heath et al., 2016; Papaoikonomou et al., 2011; Shaw et al., 2017).

We argue that both sides of the debate can benefit from an in-depth exploration of how *embodied relational* processes shape consumers’ experiences. Given that consumption happens in networks of affective and collective structures, and not in a social vacuum where individuals make discrete decisions, prior research acknowledges the importance of relational processes (i.e. Carrington et al., 2010). However, how embodied experiences shape these processes has remained in the background and we aim to bring the embodied dimension of ethical consumption practices to the fore.

From a theoretical standpoint, we draw from practice theory scholarship to investigate the embodied dimensions of ethical interactions, emphasizing the importance of bodily dispositions, material elements, and embodied senses. To highlight the interplay between embodiment and social relations, we adopt Mandalaki and Fotaki’s (2020) definition of ‘*embodied relationality*’, namely an “ethical process emerging through social actors’ mutual recognition of shared vulnerabilities, and reliance on reciprocal practical contributions that account for their actual corporeal, localised need for interdependence” (p. 752).

From a methodological perspective, we adopted an ethnographic approach, selected as an ‘embodied research practice’ (Martens et al., 2014), to observe family food practices. We selected families as a unit of analysis, for their potential to reveal everyday relational dynamics (Huff & Cotte, 2016), paying particular attention to food practices, as these allow researchers to study the embodied, routinised, and un-reflexive aspects of consumption (Neuman, 2019). Our ethnographic fieldwork with families lasted fourteen months and included participant observations of key phases of food consumption (planning, shopping, cooking, eating), semi-structured interviews, kitchen and walking-with tours, and participants’ diaries. Our findings uncover four practices which highlight the complex, often ambivalent, embodied relational states of family food dynamics: *reconciling competing embodied states; negotiating togetherness; seeking sensorial familiarity; and calibrating attention and engagement*.

This paper contributes to the attitude-behaviour gap debate on two grounds. First, we offer ‘embodied relationality’ as a theoretical framework to understand the complex ambivalent embodied states consumers experience relationally in everyday practices*.* This helps reveal neglected dimensions in most attitude-behaviour gap scholarship, namely bodily dispositions, interactions between material elements and bodies, and how embodied senses underpin (un)ethical consumption*.* Second, departing from Bell et al.’s (2021) reflections, we redefine the attitude-behaviour gap as a *disenchanted* construct, detached from the embodied nature of all social action and, as such, unhelpful for understanding its complex ethical dimensions. We thus propose alternative *re-enchantment avenues* for research, practice, and policy, outlining practical and policy contributions to support ethical consumption from an embodied relational perspective. These include recommendations for multi-sensorial interventions aimed at disrupting un-reflexivity and fostering curiosity, addressing the pre-reflexive embodied dimension of consumer choice, and cultivating embodied belonging within a local ecosystem of relationships, thereby prompting ethical practices.

The paper is structured as follows. In the next section, we review the existing literature on the attitude-behaviour gap in ethical consumption and introduce our theoretical approach. We then present our ethnographic fieldwork. Lastly, we present our findings and conclude with a discussion of the contributions of our research and suggest future avenues for research, practice, and policy.

## The Attitude-Behaviour Gap in Ethical Consumption

The Attitude-Behaviour Gap (ABG) represents one of the most prominent debates within consumer ethics research. Two main strands of research characterise this debate. On one side, studies focus on *explaining the gap* by identifying the factors that determine it, with the intent of ‘bridging’ or ‘closing’ it. On the other, research aims at *reframing the gap* through an understanding of the real-life complexities of everyday consumption. This section illustrates the most important conceptual developments on both sides of the debate, before moving on to highlight the potential of what we call an embodied relational approach to the study of consumer ethics.

### Explaining the ABG: Individual, Linear, and Rational Perspectives

Most research on the ABG relies on rational, individual-based models of decision-making (Andorfer & Liebe, 2012; ElHaffar et al., 2020; Fischer et al., 2021; Hassan et al., 2016). These approaches are grounded in an understanding of consumption as a linear and causal progression from beliefs to attitudes, attitudes to intentions, and intentions to behaviours, as outlined in the Theory of Planned Behaviour (Aizen, 1991).

The main body of research on the ABG focuses on ‘modelling the gap’ (ElHaffar et al., 2020), namely understanding the factors that determine it. These include psychological factors, such as social power (Yan et al., 2021), perceptions of time (Franco & Ghisetti, 2022), and implicit/explicit attitudes (Govind et al., 2019). Studies also consider socio-economic factors, such as price sensitivity, lack of information, and cynicism towards brands’ ethical claims (Bray et al., 2011), as well as price composition (Bürgin & Wilken, 2022). A connected body of research considers methodological issues that might explain the gap. Studies here focus on concepts such as social desirability bias, consumer self-reporting methods, lack of data on actual purchasing behaviour, and the separation in time between measuring intentions and actual behaviours (Hassan et al., 2016). A further strand of literature addresses consumers’ cognitive mechanisms for not adhering to their values, exploring how consumers ‘cope with the gap’ (ElHaffar et al., 2020). This encompasses neutralisation techniques and rational justifications for coping with the dissonance consumers experience between intentions and behaviours (Chatzidakis et al., 2007; d’Astous & Legendre, 2009; Gamma et al., 2020; Gruber & Schlegelmilch, 2014). A further strand of scholarship aims to explain the gap through a segmentation of consumers based on their levels of commitment to ethical consumption. Studies here identify different consumer segments ranging from low to high levels of ethical engagement and likelihood to display an ABG (Carrington et al., 2014; Sahelices‐Pinto et al., 2021; Yan et al., 2021).

Despite the wealth of conceptual approaches and empirical applications, linear models have been criticised for oversimplifying consumption choices and artificially isolating them from external factors. Consequently, scholars (Carrington et al., 2010; Papaoikonomou et al., 2011) call for more holistic approaches to studying the ABG, thus emphasising the collective and systemic dimensions of consumption – opening up the possibility of *reframing the gap.*

### Reframing the ABG: Collective and Multi-layered Perspectives

This alternative approach to the study of the ABG focuses on consumers’ real-life experiences, with a focus on collective structures, care responsibilities, and the uncertainty of everyday life; and has been defined as the ‘interpretive’ strand of the ABG debate (Caruana et al., 2016, p. 215). This approach is less concerned with explaining the reasons behind the gap and more with offering a nuanced understanding of consumers’ real-life experiences. We identify three key perspectives which seek to reframe the ABG through: (i) notions of compromise and uncertainty (Connolly & Prothero, 2008; Longo et al., 2019; Pecoraro & Uusitalo, 2014); (ii) the ethics of care (Carey et al., 2008; Heath et al., 2016; Papaoikonomou et al., 2011; Shaw et al., 2017); (iii) a view of consumption as multi-layered (Amilien et al., 2022; Carrington et al., 2014, 2016; Moraes et al., 2017).

The first body of research reframing the ABG stresses the need to move away from notions of consumers’ inconsistency and hypocrisy, and instead focus on concepts of compromise, uncertainty, and ambivalence. For example, Connolly and Prothero’s (2008) investigation of green consumption highlights how responsibility and empowerment (“I know that I should and can do something”) are often coupled with uncertainty (“I don’t know what is the right thing to do”). Everyday consumption is therefore not characterised by a lack of morality but by a plurality of competing moralities, becoming a site of uncertainty and paradoxes (Pecoraro & Uusitalo, 2014). Additionally, consumers are overloaded with information, which has a disempowering effect and results in confusion, scepticism, and paralysis (Longo et al., 2019). The second body of research reconceptualises the ABG as an attempt to embed an ethics of care in everyday life, studying the interactions between identity, caring commitments, ethics, and related tensions (Shaw et al., 2016). An emblematic example of this interplay concerns where consumers would not buy products they consider unethical for themselves but would do so for a family member (Shaw et al., 2017; Szmigin et al., 2009). The plurality of moral demands at play within consumption choices is particularly poignant in motherhood experiences, which are rife with conflicts, compromises, and a delicate balance between care for children and care for the environment (Carey et al., 2008; Heath et al., 2016; Papaoikonomou et al., 2011; Shaw et al., 2017).

A further strand of research adopts a ‘multi-layered’ and systemic perspective on the ABG, redefining it as a multi-faceted and collective phenomenon (Carrington et al., 2014, 2016; Coffin & Egan-Wyer, 2022; Moraes et al., 2017). Accordingly, consumers confront interwoven layers of ethical and non-ethical concerns, internal and external priorities and, as such, are forced to prioritize some over others (Carrington et al., 2014). This scholarship criticises the very concept of a ‘gap’, considering it an expression of the systemic contradictions immanent to consumer capitalism; as such it can never be bridged – only reframed (Carrington et al., 2016). Such a reframing entails moving away from the neoliberal fetish of individual agency to focus instead on the amorality of markets, thus reconceptualising the ‘ethical consumption gap’ into an ‘ethical consumption cap’ (Coffin & Egan-Wyer, 2022).

We argue that these different strands of the ‘reframing the ABG’ debate can benefit from an in-depth investigation of the role of *embodied relational* states underpinning (un)ethical consumer behaviour. Even in studies focussing on consumers lived experiences (Heath et al., 2016; Shaw et al., 2017) the role of the body in consumer practices remains in the background. Consequently, our understanding of the phenomenon is skewed towards cognitive measurements (ElHaffar et al., 2020; Fischer et al., 2021) and moral reasoning (Zollo, 2021). We thus argue for a comprehensive exploration of embodiment and embodied relational aspects of consumption practices and ethics.

## Embodiment in Consumption

Within consumer ethics scholarship, practice theories are one of the most prominent approaches that emphasize the role of the body and embodied experience (Evans, 2019; Welch & Warde, 2015). The term ‘practice theories’ refers to different, multiple, and varied approaches as there is no unified version of such a theory (Nicolini, 2012; Welch & Warde, 2015). Practice theories can be traced back to the seminal work of Bourdieu (1977) and Giddens (1984) who consider ‘practices’ as recognisable patterns of human activity that are embedded in, and shaped by, social relationships. Reckwitz (2002) offers one of the most accepted definitions of practice, distinguishing between ‘practice’ (the whole of human action, opposed to theory) and ‘practices’ (blocks of interconnected elements). Accordingly, practices are “a routinized type of behaviour which consists of several elements, interconnected to one other: forms of bodily activities, forms of mental activities, ‘things’ and their use, a background knowledge in the form of understanding, know-how, states of emotion and motivational knowledge” (*ibid.* p. 249). Importantly, bodily activities are integral to practices: from this perspective, individuals act as the ‘carriers’ of practice, i.e. practitioners of bodily behaviours, desires, and routinized understanding. At their core, practice theories hold a new conceptualisation of the body: mental and emotional states are forms of bodily phenomena, and as such emotions, cognition, perception, decision-making, and sense-making are to be considered from an embodied perspective (*ibid*.). Theories of practices are drawn to the ‘taken for granted’ and the lived experience dimension of practices, and as such are particularly interested in the body (Bourdieu, 1990).

Albeit often overlooked by mainstream approaches in consumer ethics, the body and processes of embodiment are central to the performance of practices. First and foremost, practices are performed through and by bodily processes of perception and memory: there are no practices without bodies (Wallenborn & Wilhite, 2014). Moreover, practices reveal the emotional, affective, sensorial, and social dimensions of the body, considered as a bio-socio-material nexus (Gordon et al., 2022). This relational dimension is important, as practices are shaped in, and by, social relationships: as such they are ‘embedded’ in systems of material entities (such as objects, artefacts, infrastructures) and ‘embodied’ in systems of bodily dispositions (Jacobsen & Hansen, 2021). Likewise, the emotional dimension of practices is critical, as practices are organised by emotions and their bodily expression, which shape social actors’ ethical meanings and understandings in each context (Subramani, 2022). More specifically, emotions and their bodily expressions constitute a ‘practice template’ that supports practitioners in adopting, revising, and mastering new practices (Molander & Hartmann, 2018). A focus on emotions and meanings allows a granular understanding of practices, especially when disruption of practices and adaptation to new routines occur, which can be emotionally distressing (Spotswood et al., 2024).

The centrality of the body in consumption can be further observed in processes of *embodiment,* namely integration between practices and bodies. A seminal theoretical development in this sense is Bourdieu’s concept of ‘habitus. The habitus is a repository of past experiences that becomes embodied through repetition and social interactions and spawns automated practices without conscious deliberation (Bourdieu, 1977). Through progressive embodiment, the habitus becomes a system of bodily dispositions that “generate and organise practices” (Bourdieu, 1990, p. 53). Similarly, Schatzki (2001) discusses ‘practical understanding’ as an embodied and pre-reflexive sensitivity that informs practices, a ‘knowing-how’ that is not preceded by conscious mental states. This embodied dimension allows us to conceptualise how practices crystallise into habits and routinized behaviours, conceived as ‘embodied procedures’ (Warde, 2014, p. 293). From this perspective, everyday consumption is not deliberate and mediated by thinking, but becomes embodied and pre-reflexive, being performed in sequences and repetitions, often becoming automatic, a form of ‘autopilot’ (Southerton, 2013). Mastering a new practice (i.e. mothers learning to prepare food for their children) thus involves progressive embodiment through repetition, namely incremental integration between ‘mind’ and ‘body’ until a practice becomes fluid and automatic (Molander, 2017). Additionally, mastering an everyday practice (i.e. cleaning, washing, gardening) creates an embodied sense of familiarity, whereby practices are aligned and stable, performed through embodied competencies with little to no planning or reflection (Amilien et al., 2022; Phipps & Ozanne, 2017).

We argue that adopting an embodied approach as a lens to read the ABG can extend consumer ethics scholarship on both theoretical and methodological grounds. Practices are shaped by the embodied elements of perception and memory, through which an active body ‘makes sense’ and attributes social meaning to everyday action (Wallenborn & Wilhite, 2014). A focus on the body and embodiment allows us to broaden perception beyond cognition (ibid.) and visualize emotional, affective, and sensorial elements of consumption (Gordon et al., 2022). This is particularly important in the context of food consumption, as food practices are shaped by embodied associations. Drawing on Bourdieu’s (1977) concept of habitus, and embodied sense as a precursor of all deliberative capacity, theories of practice allow us to broaden our perspective on the ABG through an emphasis on embodied sense over mental deliberation, and practical collective consciousness over private mental states (Warde, 2014; Welch & Warde, 2015).

## Methodology

### Research Design: Family Food Practices

We selected family as the unit of analysis, as family constellations represent an important focus to advance theoretical and practical understanding of consumption issues (O’Malley and Prothero, 2006). Consumer ethics scholarship is particularly interested in the ‘doing’ of families, namely how families are performed through practices of care (Huff & Cotte, 2016). Research on consumption within families includes family dynamics not only between parents and children, with a strong focus on motherhood (Heath et al., 2016; Molander, 2017; Molander & Hartmann, 2018), but also ‘senior families’, namely families with adult children and elderly parents (Huff & Cotte, 2016; Trees & Dean, 2018). We selected family food practices, such as buying groceries, preparing meals, and eating together, as these practices ‘do’ family, namely provide structure to family dynamics, contribute to creating a family identity, and maintain nurturing relationships (Trees & Dean, 2018). The consumer ethics literature has long been interested in family food practices, as the ‘meal’ is a nexus of complex consumption processes where ethical and relational aspects come to the fore (Molander, 2017; Trees & Dean, 2018; Wollendorf and Arnould, 1991). Meals are indeed performed as acts of togetherness, constructed through shared embodied moments (Wallendorf & Arnould, 1991). Given our approach to embodiment, focussing on food was particularly promising, as family food practices allow researchers to study the most ordinary, mundane, un-reflexive, and routinized aspects of consumption (Neuman, 2019).

### Participant Recruitment

Participants were recruited according to the following criteria: being part of a family (minimum two members); being (co)responsible for food decisions (planning, purchasing, cooking); owning a smartphone/tablet/laptop, and agreeing to record food practices. To gain variation in the sample (Corbin & Strauss, 2014), we asked families to self-assess their level of interest in ethical consumption as ‘low to medium’ or ‘medium to high’, recruiting three families in each category. To minimize self-reporting and social desirability bias we specified, during the screening, that we were interested in both low and high levels of commitment to ethical consumption, and reinforced our neutral stance throughout the fieldwork (Johnstone & Tan, 2015). Our sampling strategy also aimed to capture different dimensions concerning location, presence/age of children, nationality, and ethnic background (Table 1). This ensured a diverse sample to capture the nuances of ethical consumption (Carrington et al., 2014).

**Table 1:** Participants’ details

Household	Name^a^, gender, and age of family members	Engagement in ethical food consumption	Location and ethnic background
HH1	Noura (F) 44, Gavin (M) 41, Sonya (F) 7, Daisy (F) 5	Medium to high	Urban/city – Asian and White British
HH2	Rasa (F) 40, Omar (M) 45, Jonas (M) 21, Julie (F) 8	Medium to high	Urban/town – EU nationals and Asian British
HH3	Michelle (F) 57, Marcus (M) 54	Low to medium	Urban/city – White British
HH4	Claire (F) 44, Martin (M) 42, James (M) 9, Hannah (F) 6, Ellie (F) 6, Debra (F) 2	Medium to high	Rural/village – White British
HH5	Karen (F) and Rahim (M), both in their 70 s	Low to medium	Urban/city – Asian and White British
HH6	Ruth (F) 39, David (M) 40, Rob (M) 4	Low to medium	Urban/town – White British

^a^All names are pseudonyms

### Capturing the Embodied Relational Aspects of Family Food Practices: Ethnographic Approach

We adopted ethnography as an ‘embodied research practice’ for its capacity to observe and experience the embodied relationality of participants’ practices (Martens et al., 2014). Our ethnographic approach combined participant observations across key stages of food consumption (planning, shopping, cooking, and eating) with the six families over fourteen months. An ethnography of families’ food consumption is necessarily multi-sited, as it involves the home, where food is prepared and consumed, and sites for food purchasing (Evans, 2012). Our ethnographic fieldwork consisted of repeated multi-site engagements with the households, including: semi-structured interviews; kitchen tours and walking-with tours; shopping visits; and cooking and eating together with the families (see Table 2 for a summary). During multiple visits, we observed and participated in families’ food practices, accompanying family members in grocery shopping, and taking part in cooking and eating together, recorded through video and notes. Ethnographic observations were coupled with 12 semi-structured interviews (two for each family) in different seasons. All interviews were conducted in participants’ homes, to provide a safe and comfortable environment to minimise social desirability bias (Öberseder et al., 2011). Interviews were followed by ‘kitchen tours’ where we recorded (via notes, pictures, videos) the types of food products in our participants’ fridges, freezers, cupboards, and storage spaces. Kitchen inventories were undertaken due to their ability to stimulate discussion of family practices (Evans, 2012).

**Table 2:** Data collection summary

Methodology phase	Details
Ethnographic observations	Participant observations of family food practices; ‘tasting events’ and conversations; cooking and eating together; kitchen tours (multiple visits)
	Observations of phases of food consumption: planning, purchasing, cooking, eating, and disposing (multiple visits)
	Walking-with tours in supermarkets, food shops, and alternative food retail outlets, e.g. farmers’ markets, organic shops (multiple visits)
	*Dataset*: video/photographic materials: 1954 items; field notes: 30 pages
Semi-structured Interviews	12 semi-structured interviews with households (1st and 3rd visit)
	*Dataset*: interview recordings: 26 h; transcribed material: 554 pages
Food diaries	Households kept food diaries and were encouraged to share notes regarding the ‘biographies’ of their food
	*Dataset*: photographic materials: 37 items; participants’ notes

Understanding food practices from an embodied perspective requires methods engaged with the sensory and visceral dimensions of food, particularly taste and tasting (Jackson et al., 2022). The senses should be explored as interconnected, removing sight from the position of privilege granted by Western culture, and allowing other senses (particularly touch and smell) to come to the fore (Pink, 2009). For this reason, our ethnographic approach included several ‘tasting events’ (ibid.), which allowed us to explore the shared, social, and relational experiences of tasting food together. Combining conversation with an activity or a task (i.e. selecting ingredients for a dish; sharing a meal) is fruitful for provoking discussion and collecting rich multi-dimensional data on food practices (Weckroth & Närvänen, 2024). In our case, given our focus on embodied relationality, we wanted to stimulate conversations through experiential approaches, such as observing, smelling, touching, and tasting food together with the researcher.

Additionally, our fieldwork included a series of ‘walking-with tours’ (two for each family, for a total of 12 tours), where we accompanied our participants during their shopping routines to elicit further conversations. Ethnographic fieldwork on household consumption employs walking-with tours to ‘thicken’ the data with reflections based on situated actions (Evans, 2012). Participants were asked to select a familiar shopping environment, to observe their habitual food purchasing practices, and an alternative shopping environment they considered ‘ethical’, to stimulate discussion and observe practices. The walking-with method is particularly fruitful in eliciting embodied narratives of consumption: it allows researchers to capture the embodied, pre-reflexive, and tacit elements of everyday consumption, as participants and researchers move through the same space and share bodily movements (Mak et al., 2022). These shopping tours culminated in further ‘tasting events’ (Jackson et al., 2022), where the participants prepared a meal for their families and the researcher, using the ingredients (some or all) bought in the walking-with tours. This allowed the researcher to be further immersed in an embodied experience of family food practices, by sharing different phases of consumption (buying, cooking, eating, and disposing). In addition, the households completed food diaries including notes and pictures regarding their food practices, further documenting their embodied narratives of consumption (Mak et al., 2022). The final dataset included interview transcripts, video footage, pictures and notes from fieldwork, and participants’ food diaries (Table 2).

### Data Analysis

Our data analysis followed the guidelines for ‘theoretical’ and ‘latent themes’ thematic analysis proposed by Braun and Clarke (2006). Theoretical thematic analysis seeks to provide a detailed account of a specific aspect of a dataset, as opposed to a ‘data-driven’ exhaustive description of the content of the entire dataset (ibid., p. 84). For this reason, in theoretical thematic analysis coding occurs according to a specific theoretical approach, teasing out a particular feature in the data (ibid.). This approach was suitable to our study, as we were interested specifically in the embodied relational dimensions of food practices, read through embodiment theory in practice theories. We thus started with performing first-order (participant-centric) coding across the dataset. This included interview transcripts, photographic and video materials, and field notes. We then performed analytic second-order coding on the entire dataset, to generate themes. For this step, we coded the data through a practice theory lens, using the three core theoretical devices of: bodily dispositions, material elements, and embodied sense (Bourdieu, 1990; Warde, 2005). Since we were not only interested in our participants’ reflexive recollections of the embodied aspects of food practices, but also in the bodily and un-reflexive aspects of such practices, we focussed also on ‘latent themes’. Latent themes relate to the underlying elements of participant’s reflections, going beyond the semantic surface of data and what participants said (Braun & Clarke, 2006, p.84). Consequently, latent themes require interpretive work, and the analysis is already theory-informed (ibid.). We thus complemented the first-order and second-order coding with interpretive work based on our fieldwork notes and reflexivity emerging from the ethnographic engagement with the families.

## Embodied Relational Practices

The ethnographic work shed light on four embodied relational practices: *reconciling competing embodied states; negotiating togetherness; seeking sensorial familiarity;* and *calibrating attention and engagement* (Table 3).

**Table 3:** Embodied relational practices

Practice	Definitional components
Reconciling conflicting embodied states	*Bodily dispositions:* preparing, cooking, and eating meat is associated with feelings of pleasure, enjoyment, and togetherness; however, meat production and intensive farming is associated with bodily dispositions of disgust and emotion of guilt
	*Material elements:* families’ meat consumption is associated with material elements (i.e. barbecues), specific environments (i.e. camping; eating outdoors) and social dimensions (i.e. eating with extended family; celebrations with friends)
	*Embodied sense:* vegetarian and vegan consumption practices are associated with an embodied sense of constraint and awkwardness; however, repetition and interaction integrate practices until they become natural
Negotiating togetherness	*Bodily dispositions:* enjoyment, pleasure, and satisfaction connected to shared and relational food practices, with family members, nature, or community (i.e. foraging walks, homemade jams); pleasurable memories connected to childhood food practices
	*Material elements:* connection is fostered through material elements (i.e. the garden; the local business) and practical knowledge (i.e. traditional recipes)
	*Embodied sense:* families gather an embodied sense of connection, togetherness, and belonging through shared sensorial food practices; although some practices are considered limited and ‘escapist’, they represent complex renegotiations between identities, belonging, and consumption
Seeking sensorial familiarity	*Bodily dispositions:* familiar foods afford bodily dispositions of comfort, pleasure, and security, especially in children; familiar shopping environments afford bodily dispositions of safety, convenience, and efficiency
	*Material elements*: appearance, shape, colour, smell, and taste of food build un-reflexive preferences for some products over others, competing with the ethical but immaterial dimensions of food
	*Embodied sense:* comfort afforded not only by familiarity; but also bodily sensations of ‘feeling trapped’ and ‘constrained’ in rigid routines
Calibrating attention and engagement	*Bodily dispositions:* large shopping outlets generate bodily dispositions of discomfort and sensory overload; smaller ‘ethical’ shops facilitate bodily dispositions of openness and curiosity
	*Material elements:* supermarkets are associated with fast, routinised, and efficient shopping, where participants follow well-known routes; smaller shops present fewer items and are associated not only with higher quality and ethical credentials but also a lack of convenience
	*Embodied sense:* habitual shopping environments afford pre-reflexive efficiency, repetition, and mastery of practices, but hinder curiosity. Novel shopping environments can stimulate curiosity and reflection

### Reconciling Conflicting Embodied States

A key embodied relational practice of family food consumption entails reconciling conflicting embodied states, namely co-present and competing desires, bodily dispositions, and emotions. This was particularly evident in the practice of meat consumption. All the families in our fieldwork were engaged, to different extents, in reducing or eliminating meat from their diets. The most committed family was Rasa and Omar’s, and their two children, Jonas (21) and Julie (8). The family displayed a series of interconnected influences, whereby Jonas was guided by his mother’s veganism, whilst her father’s meat-eating habits affected Julie. After being vegetarian for several years, Rasa became vegan four years before our fieldwork, encouraging her son Jonas to follow her:

Rasa: I became vegetarian first and then I started to speak with my son about it, the animals and this and that and he tried for the first time, he was vegetarian for a good two weeks and then you went back to eating chicken?

Jonas: Yes, because it tasted good. […] I guess before, when you just start out and you’re not sure what you’re doing, you’re still at that experimental stage, you do kind of feel intimidated and maybe a little constrained choosing stuff so easily and you still have to think about it. But once it becomes a part of you, you don’t care and even when people ask you “what do you eat?” I say, “I eat everything except those things.” And after a whilst it just becomes natural. [Interview]

The family was committed to this lifestyle despite its effect on their social life. They had, for example, stopped being invited over for family meals by some of their family members, as explained by Rasa:

Rasa: We used to meet one of our family for barbecues and I noticed that they don’t invite us any more just because they don’t know how to cater for us. Like, if they would come, we would offer them vegetarian food or vegan food but we don’t mind, but they probably don’t know how to cook for us, you know? But sometimes you can bring your food over but you feel awkward sometimes. [Interview]

Here several competing embodied states co-exist in shared eating practices. Jonas expressed not only an embodied feeling of pleasure related to the act of tasting and eating chicken, but also feelings of care for the animals, which explains his struggle to reduce meat and his zigzagging between meat-eating and veganism. An embodied sense of feeling ‘intimidated’ and ‘constrained’ by the limited food options afforded by a vegan diet accompanied these emotional states. A similar complexity of ambivalent emotional states was reported by Rasa, who expressed a desire to participate in barbecues with her extended family, which clashed with her commitment to a vegan diet, generating feelings of ‘awkwardness’ arising from the conflicting food habits. However, through practice and repetition, these embodied states were reconciled in an increasing feeling of naturalness and mind–body integration, that transformed their vegan diet to the point that *‘it becomes a part of you’*.

Another family prominently committed to the reduction of meat was Claire and Martin’s. Claire became vegetarian when she was 13 years of age and has oscillated between vegetarianism and meat consumption ever since. Martin had been vegetarian since he was 11 years of age, despite being brought up in a family that consumed meat every day. However, after having children, and due to his work in the US where meat consumption is prevalent in work settings, Martin started to eat meat again. Their first son, James, was 9 years old at the time of our study and had been vegetarian for a few years when he was younger. They also had three young girls, whom they described as ‘fussy eaters’. At the time of our fieldwork, the family’s meat consumption practices were in flux:

Claire: I think a lot of meat is produced horribly in this country so I think that’s partly why we try not to eat so much as well really. […] But then I think we started eating meat as well because of struggling with the children, with them being very fussy as well.

Martin: Yes, but James was vegetarian for a long time.

Claire:Yes, he was vegetarian but I can’t really remember why we started eating more meat.

Martin: The girls.

Claire: Yes, exactly. So we were a bit like... they would eat fish so we would get fish and then I guess it started to creep in. But I think, because I don’t really like cooking meat, you’re much more likely to cook meat than I am as well because I worry about it.

Martin: It tends to be more on a Sunday and it tends to be a roast. […] We like eating outside. I think it’s a really nice thing to do and it feels... (he pauses) also, we normally go camping in the summer so it’s getting ready for that but I like to barbecue stuff and cook outside. […]

Claire: I suppose it’s like having meat as the special dish, isn’t it, once a week to have it if friends are round, as a roast.

Martin: I always feel a little bit guilty.

Claire: Do you? Yes, I don’t, I enjoy it.

Martin: Part of me feels a little bit like ‘mmm…’ (he gestures dissatisfaction). [Interview and fieldwork notes]

This exchange shows several dynamics at play. Here the two partners engaged in relational reflexivity concerning their shared meat consumption practices. Both associated the production of meat, especially in intensive farming systems, with disgust and guilt, stressing the embodied state of animals ‘stuck’ in cages as ‘awful’. However, they engaged in this eating practice to accommodate children’s preferences, work patterns, and broader social influences, such as holidays with friends. Whilst a conventional ABG lens would read these exchanges as an example of the discrepancy between Claire and Martin’s attitudes and behaviours, an embodied relational perspective shows us the co-presence of different embodied states, which they negotiate individually and relationally. From an embodied perspective, Claire expressed not only a sense of disgust and dislike towards buying and cooking meat, which she avoided, but also bodily dispositions of pleasure and enjoyment related the physical act of eating and tasting meat. For Martin, conversely, bodily dispositions of pleasure and excitement were related to the material elements of preparing meat (enjoyment gained from roasting meat on a barbecue, eating outdoors), its embodied relational aspects (sharing with friends), and evoked pleasurable memories (i.e. summer holidays). However, these positive embodied and emotional states co-existed with a visceral tension related to the act of eating meat, which generated guilt and resistance. Claire and Martin reconciled these conflicting embodied states both individually, in their personal consumption (i.e. Claire would not buy or cook meat, but would eat it), and relationally (Martin would roast meat for his family and friends but would not enjoy eating it). Moving the focus from an attitude-behaviour discrepancy to an exploration of competing and co-existing embodied states permits a more nuanced understanding of family food practices.

### Negotiating Togetherness Through Food

To different extents, all our participants engaged in food practices such as supporting local food producers, pick-your-own schemes, foraging for food, and growing fruits and herbs. The food diaries and ethnographic observations indicated participants felt an embodied sense of pleasure, connection, and enjoyment associated with these food practices, which were incorporated at the family level to cultivate belonging and togetherness (fieldwork notes). In the reflexive accounts shared in interviews, these practices were depicted with affectionate tones that demonstrated participants gathered an embodied sense of satisfaction and belonging from them (interviews).

**Fig. 1:**
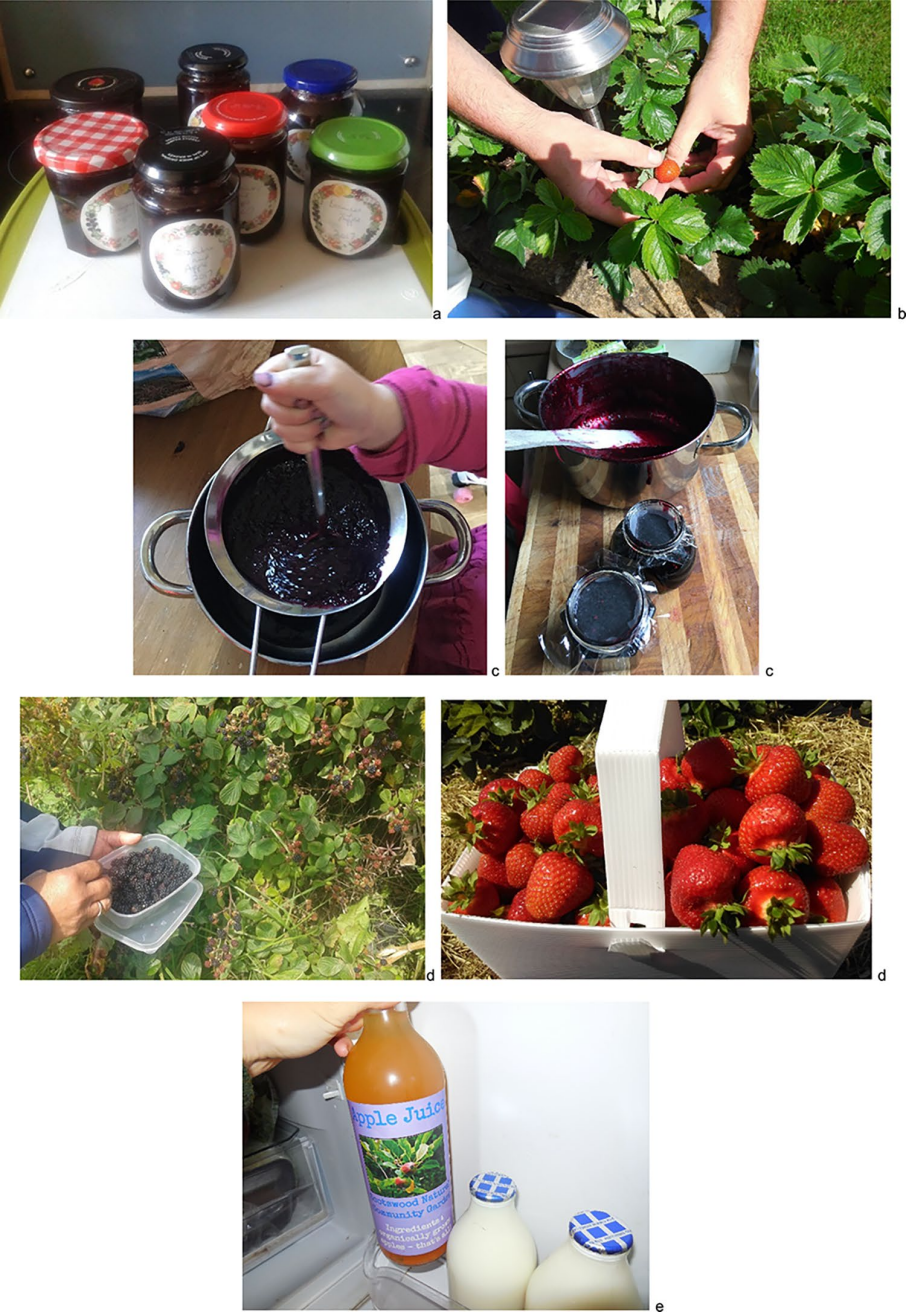
**a** Homemade jams (Karen’s food diary). **b** Michelle and Marcus’s strawberries (fieldwork picture). **c** Preparing homemade jam with children (Claire’s food diary). **d** Foraging fruits and pick-your-own schemes (Karen’s and Rasa’s food diary). **e** Karen and Rahim’s local milk and juices (fieldwork picture)

Karen used to forage berries from wild bushes. She talked about how she loved going on walks to pick blackberries in summer and experimenting with her grandchildren to make jams (Fig. 1a). Similarly, Claire and her children would often make jams together (Fig. 1c) and Rasa would go to pick-your-own farms (Fig. 1d). However, family members often had to renegotiate the sense of togetherness and belonging generated by these practices with an understanding of these practices as partial and temporary. Despite the enjoyment and pleasure family members reported and displayed when discussing these activities, they also considered them limited and occasionally ‘escapist’ (food diaries). For example, Michelle and Marcus grew small amounts of food in their garden, for enjoyment and sensorial engagement with the outdoor space. During fieldwork, they proudly showed their garden to the researcher, affectionately moving the strawberry plant to show the newly grown strawberries (fieldwork notes—Fig. 1b)—which they reported to be “*much tastier than the ones you buy in the shops”* (Michelle’s interview). Michelle was particularly trusting of home-grown food, even more than food sold in farmer markets; however, she experienced tensions related to the enjoyment of growing her own food, her trust in home-grown food, and her limited ability to do so, due to time and space constraints:

Michelle: I wouldn’t go to a food market in Jesmond or whatever. But I would if it was that kind of... just people growing their own stuff. I’d always go to allotment shops. I like the idea of people growing their own vegetables. I’d love to grow my own vegetables more. If there was a choice, I’d really like to grow more myself.

Michelle acknowledged the tensions between the enjoyment and trust she felt for home-grown food and feelings of being constrained and ‘not having a choice’ to grow more in her garden. Similarly, Karen and Rahim were keen to support local production and purchased fresh juices and milk from a local independent milkman (Fig. 1e). They reported they were committed to buying from the local milkman despite the price being three times higher than that of supermarkets:

Rahim: We get our milk from a milkman who comes every morning, and he … obviously we pay more for it, we do realise we can get it from elsewhere, but we are paying such a ridiculous amount because supermarkets are taking their livelihood because they are big….

Karen: They Bully Their Suppliers.

In this quotation, the choice of buying juices and milk from a local milkman and thus cultivating belonging and solidarity with the local community, was negotiated with the perception of the milk price as ‘ridiculous’, signalling conflicting feelings around the higher price paid.

Negotiating togetherness through food was even more apparent when participants spontaneously shared recollections about childhood memories. Rasa, for example, had Lithuanian origins and spent most of her childhood summers in her grandmother’s village. She cultivated this sense of belonging to her Lithuanian origins by cooking recipes from her homeland whilst living in the UK. She expressed how shared cooking of traditional recipes was a way of fostering her connection with her brother:

Rasa: I have been raised in Lithuania and I spent my summers at my grandma’s village so I have that knowledge about local foods and the seasonal foods and I know what comes in season. […] I do cook but not as often and I would like to try some Cepelinai [traditional soup], but it’s hard work. When I go to Lithuania, my brother is a very good cook, so, I say “brother, we’ll make it.” You know, together as a team. So, we did it, I peeled potatoes and then he came back from work and we cooked together. [Interview]

Here the connection is expressed in the embodied sense of ‘being a team’ and sharing knowledge about traditional recipes. Preparing the Cepelinai soup represented for Rasa a complex renegotiation of her identity as an immigrant, sense of belonging to her family of origin, connection to her brother, as well as nostalgic connection to local Lithuanian food.

Similarly, Noura, who had Indian origins, shared how she would often eat in local Indian restaurants in her neighbourhood, as it brought back childhood memories of feelings of togetherness and connection to her family:

Noura: As a family, growing up food was really important, […] eating together as a family was really important. We used to do that a lot for family occasions, meals out, birthdays, just generally. We lived in London and my dad’s Indian so we ate a lot of Indian food that was really good quality and really cheap and really local. It always had a very big cultural factor, even with extended family and when people visit. I really liked that. [Interview*]*

Noura’s adult eating practices and love for local Indian restaurants were informed by the embodied feelings of connection and togetherness experienced as a child in such food places. These spontaneous childhood recollections show how embodied relational aspects are a central driver shaping food practices, as our participants skilfully renegotiated their embodied sense of connection with their families through childhood memories about food that then became food practices in adulthood.

This is relevant to our understanding of the ABG phenomenon. Much previous research on the ABG focuses on the aspects of consumption that are intentional, reflexive, and value-based (El-Haffar et al., 2020). However, an embodied relational perspective reveals that consumption revolves around complex negotiations concerning identity, connection, togetherness, and belonging, either with current family members (i.e. Karen’s grandchildren), nature and outdoors (Michelle and Marcus’ Garden), the local community (Karen and Rahim’s milkman), or one’s family of origin (as in Rasa’s and Noura’s examples). These negotiations constitute part of the complex emotional landscape of consumption, which is often missed in research on the ABG. This invites us to refocus on the latent embodied relational aspects underpinning everyday consumption, rather than on intentional individual choices.

### Seeking Sensorial Familiarity

Through our ethnographic fieldwork, we found that a central embodied relational practice shaping families’ food consumption was seeking sensorial familiarity with food items and shopping environments. This was particularly evident in children’s behaviours. Children’s personal preferences play a key role in shaping families’ food practices, sometimes in an opposite direction to the ethical preferences of their parents. For example, Claire and Martin’s household was occasionally keen to buy high-quality and local farmers’ products, which they felt were healthier and more ethical than supermarket alternatives. However, during our tasting events, it became apparent that children’s familiarity-seeking dispositions limited parents’ choices. During one of our fieldwork visits, Claire and Martin were joined in our shared tasting event by two of their children, James (9) and Ellie (6). Whilst trying and tasting some Parma ham, the family members engaged in shared reflections on their food habits:

Martin:It’s the sort of thing we might have at Christmas or something, isn’t it?

Claire:Yes, more like a treat. […]

Martin: The kids eat ham but not Parma ham.

Claire: No, they don’t eat Parma ham. They just eat cheap ham, don’t they? […] like sliced ham for sandwiches, that kind of thing. So no, we wouldn’t really... that’s more like a treat, isn’t it, Parma ham. (Talking to Ellie) Have that bit darling. Did you like that? […] You don’t like that ham? Why not?

Ellie: I tried this ham before and I don’t like it.

Researcher: What’s the ham you like?

Ellie: I like the other kind of ham, the pinky one.

Claire: Because this is probably a bit saltier, isn’t it, than I guess the one that you’re used to. [Tasting event]

Here Claire invited her child to try high-quality Parma ham, which the family did not consume regularly and was considered a treat. When Ellie tried the food, she expressed bodily dispositions of dislike (fieldwork notes), which Claire immediately interpreted as related to Ellie being ‘not used to the saltiness’ of Parma ham. Several similar occasions occurred during our fieldwork. Claire recalled an instance in which she bought fresh carrots from a local farmers’ market. As James, her son, was familiar with the appearance, shape, and taste of conventional vegetables, he struggled to eat local farmers’ carrots:

Claire: James, I bought him… […] some carrots from the market that still had the carrot tops on so they were really nice and fresh. He didn’t like those. I think he was so used to eating quite flavourless vegetables from [a local wholesaler], which is a bit sad really. [Interview]

Similarly, when the family experimented with a local organic vegetable box scheme, James was “*really fussy about his perfect green apples with no blemishes*” (Claire’s interview), and his parents accommodated his preferences by buying perfectly looking apples at a local supermarket.

Here these instances show how the embodied state of familiarity afforded by food appearance and taste led children to develop un-reflexive preferences for specific products – the ‘pinky’ ham; the ‘perfect’ green apples. This familiarity-seeking hindered the family’s ability to adopt healthier and more ethical choices. The conflicting preferences of family members, and the connected feelings of frustration, were often a discussion theme in our informal conversations whilst preparing and eating food with the families (fieldwork notes).

Although these dynamics were more prominent in parent–child interactions, they also occurred between adults. For example, Ruth and David were keen to buy organic products on both health and ethical grounds. However, their sense of familiarity and sensorial preferences for conventional products often hindered consumption of organic foods, as in this case relating to milk:

Ruth: I would prefer to buy organic milk but David and Rob don’t like the taste or the texture or something?

David:I don’t know. I’ve always been quite funny when it comes to milk. I can taste the differences in milk really easily whereas you clearly can’t […] and there’s something I don’t like about the organic milk. It just doesn’t taste right. I don’t know if it’s because I’m not used to it and it’s just a difference. I was so chuffed though when Rob had a drink of milk and didn’t like it [laughter] […] because Ruth was like, “It’s no different. It’s just the same,” and then Rob was like, “This milk is horrible.” I was like… “yeah. It’s definitely different”. [Interview]

Seeking familiarity played an important role in shaping our participants’ consumption practices, both in children and adults. We observed another familiarity-seeking practice in relation to retailers’ material characteristics. In our walking-with tours, participants displayed a body language conveying comfort and experience when navigating the aisles of familiar retailers, expressed through walking with confidence along what seemed to be well-known paths, immediately finding their desired products, and not hesitating when moving around the store (fieldwork notes). This embodied familiarity sometimes translated into a difficultly in altering their habitual place of shopping, even when our participants admitted they were dissatisfied with some of the product offerings or overall policies. For example, during our first visit, Karen reported conflicting states regarding her habitual retailer. She was passionate about ethical issues and committed to the Fairtrade certification, however, her habitual retailer had just announced their withdrawal from such scheme. This prompted some reflections with her husband, Rahim:

Rahim:Well, your supermarket that has just withdrawn from Fairtrade… […]

Karen:Well, I might have to change my supermarket, honestly. […]

Rahim: You say you like [retailer’s name] because they provide…

Karen:(she interrupts him) I’m used to it, I suppose. I’ll have to change. [Interview]

During our fieldwork, Karen boycotted the retailer to support the Fairtrade label. She also tried to contact the local branch manager and her MP. This effort lasted three months. However, during our last visit, Karen reported: “*I stopped going to [retailer’s name] for about three months and then went back again, because I was very familiar with it.*” Here, the embodied sense of familiarity and comfort afforded by the retailer constituted a stronger driver than her passionate commitment to the Fairtrade label. Similarly, Ruth was a subscriber to a box scheme that delivers ingredients and recipes for pre-planned meals. At the beginning of her subscription, the company sourced most of its products from local and organic suppliers, often certified with the RSPCA and Organic labels. However, as the company expanded, the commitment to certified standards diluted and the company adopted in-house certifications. This did not prompt a change in consumption habits in Ruth’s household:

Ruth: At least in the early days, they tried to source things from independent growers and producers as well. I don’t notice that so much now. Because they used to send… they would send you things with all of this labelling on, so you knew it had come from somebody independent. But now they’ve just branded everything [with the box scheme company]. All the little packets of spices or honey or condiments that you receive from them is labelled [with the box scheme company], which is a bit of a shame. But it’s really convenient. [Interview]

Albeit confessing to being concerned with a potential dilution of ethical standards in the company’s choices, Ruth’s primary considerations of familiarity and convenience remained critical in preserving existing family practices. However, in informal conversations during walking-with tours, as well as in interviews, the familiarity revealed also a constraining side, whereby families expressed feelings of being ‘trapped’ by it. For example, Noura and Gavin repeatedly reported being dissatisfied with their food habits. The lack of time, resources, and creativity, as well as the need to accommodate their children’s food preferences, drove them to establish a rigid routine in terms of food choices, which they felt unable to escape:

Noura:When the kids were little, we didn’t try lots and lots of different food. They were quite set on what they eat […] They totally drive our food decisions.

Gavin:But they are trying more new things because they’re a bit older, which is good for us because otherwise I get bored eating what they eat. […] Eating becomes functional rather than for enjoyment. When it’s with the kids it does, yes. […]

Noura:But it’s always the same food and it’s really... I feel incapable of looking. So, Gavin went to the supermarket yesterday, we had nothing in and I knew because what he’d bought is what I would have chosen. There was nothing different. It’s what we buy. [Interview]

Noura described their food as ‘*convenient, easy, boring*’ and their routines unconducive to making new, or different, choices in terms of ethical shopping. We thus observed how an embodied sense of familiarity generated both comfort and discomfort, as families felt both eased and constrained by it. These instances, related to familiarity with sensorial aspects of food and the material characteristics of retailers, demonstrate how an embodied relational perspective can broaden our understanding of family food practices. An ABG approach would emphasise the discrepancy between families’ attitudes towards ethical food products and shopping outlets (i.e. locally grown products; ethical certifications) and their actual behaviour. However, from an embodied perspective, we observe how a sense of ‘ontological security’ (Giddens, 1984) afforded by familiar foods and retailers shapes a pre-reflexive sensitivity towards certain choices. Whilst this security ensured the stability of practices, this locked participants into (often uncomfortable) routines.

### Calibrating Attention and Engagement

Lastly, we found that our participants calibrated their attention and engagement in response to different shopping environments, exhibiting different embodied states depending on their situated and relational position. In practice theories, the external environment constitutes a key factor shaping blocks and sequences of practices (Reckwitz, 2002). We observed this differentiation both first-hand in our shopping trips (fieldwork notes) and reflexively represented in our participants’ words (interviews). Most of our participants perceived supermarkets as a place for efficient, fast, routinized, and un-reflexive shopping, and thus unconducive to paying attention to products bearing ethical credentials. They thus traversed supermarkets whilst paying little attention to the surroundings and displayed little engagement with product characteristics. Ruth and David, for example, were keen on buying organic foods and could afford to pay for them. However, several barriers remained. For David, the supermarket did not represent a good place to engage with ethical credentials of food:

David:I think even just the way that the supermarkets are set up: they’re trying to get you to buy certain things; there’s certain ways that they want you to walk around; there’s certain brands that they want you to see that are at eye level. […] Sometimes you go in the supermarket, and you just want to be out of the supermarket [laughter]. You’re just kind of grabbing things and you’re not really thinking and concentrating on what you’re doing. You’ve just got a list of what you need, and you just want to be in and out as quick as you can. […] So, you’re not really concentrating on getting the best things. […] We think it’s really important [organic food] but as I say when you’re rushing around the supermarket trying to get out, maybe not so. Maybe getting out of the supermarket is more important. [Interview]

David explained how his willingness to buy organic products was hindered by the material characteristics of supermarkets, which generated embodied states of discomfort and rush, to the point that ‘getting out of the supermarket’ became more important than ‘buying organic products’. We observed this dynamic in our shopping tours with other families too, where participants appeared to follow well-known paths in large supermarkets, engaging with only a few products, searching for familiar items they had often written down on lists, paying limited attention to their wider surroundings, and displaying a body language conveying an embodied sense of ‘rushing around’ (fieldwork notes).

Poignantly, Claire presented us with a distinction between supermarkets, considered places for efficient food shopping, and local independent shops, where instead she felt less subject to sensory overload and thus more able to engage with the ethical characteristics of products. Commenting on a locally produced honey, she noted:

Claire: It depends on what kind of shopping you would do, doesn’t it? If you were doing a food shop, I’d probably just walk past this, even if it was cheap. But if I was in a different environment, I would probably choose to... if I was in a little farm shop and I saw some stuff like this, I’d be more tempted because there’d be less of everything else. You weren’t going shopping for food, you’re in a more bespoke environment that’s focussed on... [ethical products], but normally I probably wouldn’t [buy it]. [Interview]

We consistently found this differentiation in our walking-with tours in ‘alternative’ and ‘ethical’ shopping outlets chosen by our participants. Here participants engaged with their surroundings, displaying an embodied sense of curiosity and body language conveying openness (fieldwork notes). Overall, the external environment played an important role in shaping our participants’ embodied senses: in large retailers, participants showed and expressed feelings of ‘racing around’ and ‘wanting to be out quickly’, reporting sensory overload and calibrating attention just to familiar food items. In alternative and smaller food outlets, instead, participants showed openness and ability to engage with food with curiosity, thus paying more attention to the ethical characteristics of products. This has important implications for our understanding of the ABG. Whilst much previous ABG research does not emphasise the material affordances of shopping outlets, an embodied approach allows us to understand how bodies interact with products in different ways depending on the material characteristics of place, with important consequences for (un)ethical consumption.

## Re-enchanting Everyday Consumer Ethics: A Research and Policy Agenda

We now tease out the main contributions emerging from the findings, suggest avenues for future research, and recommend interventions from an embodied relationality perspective.

### Contribution to ABG Scholarship

This paper contributes to different strands of the ABG debate. First, much ABG scholarship aimed at ‘explaining the gap’ considers the individual factors that determine it (Bürgin & Wilken, 2022; Govind et al., 2019; Yan et al., 2021) or consumer justification mechanisms (d’Astous & Legendre, 2009; Gamma et al., 2020; Gruber & Schlegelmilch, 2014). Such approaches privilege rational and linear models of individual choice, emphasising planning and intention (Carrington et al., 2010; Hassan et al., 2016). Conversely, an embodied relational perspective highlights the central role played by *pre-reflexive embodied sense* in shaping consumer practices. This was particularly evident in terms of the ontological security (Giddens, 1984) afforded by familiar food products and shopping places which shape consumer ethical (or unethical) practices. In this way, a focus on embodied relationality uncovers neglected dimensions, hidden in most ABG scholarship aiming at ‘explaining the gap’, namely bodily dispositions, material elements-bodies interactions, and embodied senses associated with consumption (Warde, 2005). This is important, as exploring the embodied relational aspects of consumption allows us to better understand the ambivalent states and complex uncertainties that explain the persistence of unsustainable practices.

A further strand of ABG scholarship focuses instead on ‘reframing the gap’, stressing the importance of consumer uncertainty (Connolly & Prothero, 2008; Longo et al., 2019; Pecoraro & Uusitalo, 2014) and multiple caring commitments (Carey et al., 2008; Heath et al., 2016; Papaoikonomou et al., 2011; Shaw et al., 2017). However, whilst emphasising consumers lived experience, this scholarship still privileges the cognitive dimensions of consumer ethics – e.g. confusion due to knowledge overload (Longo et al., 2019) or reflexive dimensions of consumer responsibility (Shaw et al., 2017). The centrality of the cognitive dimension is reflected in methodological choices, as these ABG studies often adopt interviews where participants reflexively articulate their views (Heath et al., 2016; Shaw et al., 2017). Additionally, whilst previous studies emphasise the dilemmas and uncertainties that lead consumers to paralysis (Longo et al., 2019; Pecoraro & Uusitalo, 2014; Shaw et al., 2016) here we stress the embodied relational and emotional dimensions of these dilemmas and tensions. This is important for understanding the complex dynamics at play within consumer practices, from a holistic embodied view of consumption.

Leveraging our empirical insights, we argue, consistent with Bell et al. (2021), that the dominant epistemological paradigms of objectivism and positivism have hindered researchers’ ability to understand complex social phenomena, reducing them to obscure rational and measurable concepts. Consequently, Bell et al. (2021) suggest much research has become *disenchanted*, namely separated from the relational nature of phenomena, which are inherently embodied, and, in turn, ethical and political. We respond to these reflections to propose a critique of the ABG as a *disenchanted* construct, namely detached from the embodied nature of all social action and, as such, with limited potential to explain its complex ethical, social, and political dimensions. This aligns with critical strands of the consumer ethics literature, which critique the concept of a ‘gap’, considering it the product of a neoliberal ideology that fetishizes individual agency and downplays the contradictions immanent to capitalist economies (Carrington et al., 2016; Coffin & Egan-Wyer, 2022). We argue that, whilst such critiques of individual responses to capitalist contradictions are important and necessary, collective responses will benefit from a keen understanding of the embodied relational processes that support them. We thus extend critical consumer ethics scholarship by arguing that the ABG is not a methodological issue (how to best investigate the gap) or a policy issue (how to bridge the gap) but an epistemological issue (a disenchanted construct). This is significant, as we argue that scholarship attempting to ‘explain’ or ‘reframe’ the ABG without an embodied relational lens is *sharpening the wrong tools*. Specifically, increasing the sophistication of models or expanding the consideration of contextual factors does not make up for the lack of an embodied relational understanding of food practices and consumer practices. Departing from this, we propose four *re-enchantment* avenues (Bell et al., 2021) for future research and interventions to deepen engagement with an embodied relationality perspective (see Table 4).

**Table 4:** Re-enchanting consumer ethics from an embodied relationality perspective

Consumer practices	Contribution to ABG literature	Future research avenues	Policy and Managerial Implications
Reconciling conflicting embodied states	Existing scholarship emphasises *cognitive dimensions of consumer uncertainty*, such as knowledge overload (Longo et al., 2019). We expand this by highlighting conflicting *embodied states* and their* constellations of bodily dispositions and material elements*	*Avenue 1.* Engage in methodological innovations not only able to accommodate embodied and pre-reflexive dimensions of consumer ethics, but also consider multiple human and more-than-human embodied entanglements	Design interventions able to address the pre-reflexive and pre-verbal dimension of embodied and relational consumer choice, likely requiring inter- and trans-disciplinary efforts
Negotiating togetherness	Existing scholarship focuses on *reflexive dimensions of care and togetherness*, such as ‘care about’ and ‘care for’ (Shaw et al., 2017). We complement this by giving visibility to the *embodied and pre-reflexive negotiations of care and togetherness*, including emotional and bodily dispositions	*Avenue 2.* Explore how consumer ethics scholarship can further deepen our understanding of the role of familiarity, affectivity, and inter-corporeality in decision-making. Investigate what individuals’ embodied experiences of collective, structural, and institutional dimensions of consumption are	Design interventions that foster connection with local businesses and community actors, harnessing embodied togetherness to foster belonging to a local ecosystem of relationships and thus prompt ethical practices
Seeking sensorial familiarity	Existing research privileges *rational and linear models of individual choice,* emphasising planning and intention (Carrington et al., 2010; Hassan et al., 2016). We show instead the central role of *pre-reflexive embodied sense of ontological security* afforded by familiar products and places which drives practices	*Avenue 3.* Future research needs to engage with all the senses, including touch, taste, and smell, to further understand how familiarity with consumption practices is developed from an embodied perspective and how curiosity and experimentation might be fostered	Trialling interventions that disrupt un-reflexivity. For example, multi-dimensional installations which provoke curiosity, wonder, and consideration of ethical-environmental concerns. Multi-sensorial experimental interventions can also stimulate children’s curiosity for healthy and natural-looking food
Calibrating attention and engagement	Critical consumer ethics scholarship emphasises the *constraints individuals face in the market* (Carrington et al., 2016; Coffin & Egan-Wyer, 2022). We enrich these insights by unpacking how *bodies interact with such material constraints*	*Avenue 4.* Employ embodied relational methods to investigate what embodied relational states are more conducive to destabilization, misalignment, and reconfiguration of constellations of practices	Shift the focus from micro-level interventions targeting the individual to systemic interventions targeting multiple actors whilst taking into account an embodied relational perspective

### Contribution to Consumer Ethics Scholarship

A vast literature now exists regarding ethical consumption practices and ethical decision-making. Whilst we acknowledge that notions of embodiment are sometimes mentioned in different strands of the consumer ethics literature, they are seldom empirically investigated, analysed, and theorised. For example, a systematic review of the literature on consumer ethics analysed 155 articles on the topic, across twelve disciplines, including, amongst others, social and political science, philosophy, business and management, marketing, and psychology—the words ‘body’ or ‘embodied’ were not mentioned once (Carrington et al., 2021). Even in studies that explicitly call for more attention to emotions and intuition, the role of the body in consumer ethics is largely forgotten (Zollo, 2021). Yet, our empirical study demonstrates that using the body as a methodological and conceptual tool (Wallenborn & Wilhite, 2014) generates novel findings on the complex negotiations of everyday ethics consumers are immersed in. An embodied relational approach expands our understanding of the complex, often ambivalent, nature of consumer ethics, and shows how consumers reconcile, renegotiate, and calibrate competing embodied states and ethical associations. We thus argue that not only to be able to generate a thick but also granular understanding of consumer ethics, bodies and their complex ethical relations should be centre stage. This is important, as the field of consumer ethics is at the forefront in the ‘grand ethical challenge’ of our times, namely reconciling consumption with planetary boundaries (Böhm et al., 2022). An embodied relational approach can inform policy aiming at systemic change; this is especially true in the case of ambitious policymaking that is moving away from interventions focussed on individual behaviour towards a focus instead on policy interconnections and systems thinking (EU Policy Lab, 2025).

### Contribution to Practice Theory Scholarship

The work of first-generation practice theorists such as Bourdieu (1977, 1990) highlight how the body mediates the lived experience of social actors in different contexts. However, recent practice theory scholarship on ethical consumption has been more concerned with the role of materiality (i.e. objects, technology, infrastructures), whilst downplaying the embodied aspects of practices (for a review, see Jacobsen & Hansen, 2021). In other words, scholarship has been more concerned with how practices are ‘embedded’ in socio-material infrastructures rather than ‘embodied’ (ibid.). Examples include practice theory studies focussed on the ‘objects, meanings, doings, and places’ of luxury consumption (Moraes et al., 2017) and the routines, artifacts, and competencies underpinning family food consumption (Huff & Cotte, 2016; Trees & Dean, 2018). Whilst these studies recognise that immaterial and embodied elements shape consumption practices, they do not explore and unpack the embodied dimension. Similarly, studies focussed on embodiment leave room for a deeper exploration. For example, Molander’s (2017) study on motherhood and consumption practices explored learning as an embodied experience, focussing on the dimensions of skills and positioning, thus leaving room for a deeper exploration of relational embodiment. We thus contribute to practice theory scholarship by responding to Jacobsen and Hansen’s (2021) call for reconnecting with first-generation theorists’ attention to the body and thus conducting more research on the embodied relational aspects of consumption, particularly embodiment through social relations.

### Re-enchantment Avenues: Research and Policy

#### Avenue 1

From a methodological perspective, re-enchantment can be practiced with methods favouring the embodied and pre-reflexive dimensions of consumer ethics. Our methodological contribution consists of a comprehensive participatory method combining different methodological advancements in the field of food practices, such as activity-based discussion (Weckroth and Narvanen, 2024), tasting events (Jackson et al., 2022), and walking-with tours (Mak et al., 2022). This generated rich, embodied multi-dimensional data. We call for future research to expand this approach and engage in inter- and trans-disciplinary efforts, for example with psychology and neurosciences. Developments in the field of neuro-gastronomy are promising to understand the connections between the senses, desire, and consumption practices, and explore how edibility is relationally constructed (Jackson et al., 2022). Additionally, we invite future research and policy efforts to expand reflections on embodied relationality to ethical encounters with more-than-human bodies and materialities. Given that questions of ethical consumption bring to the fore the issues of biodiversity loss and co-habitation of the planet, we need to develop our understanding of entanglements ethics (Dale & Latham, 2015). This aligns with the policy approach of ‘One Health’, highlighting the interconnections between different bodies (humans and more-than-human) and ecosystems at large (FAO et al., 2022).

#### Avenue 2

Consumption is inherently relational, intertwined in a complex web of social relationships, ties, and caring commitments (Carrington et al., 2016; Shaw et al., 2017). We invite future research to further explore the embodied relational dimension beyond the family unit, for example exploring corporeality and affectivity in collective and institutional consumption spaces. From a policy and managerial perspective, this could translate into interventions fostering connection with local businesses and community actors, harnessing embodied togetherness to foster belonging to a local ecosystem of relationships and thus enhancing ethical choices.

#### Avenue 3

Most consumption policies seeking to foster ethical consumption are ‘mind-centred’ and ‘body-absent’ (Wallenborn & Wilhite, 2014). This narrows perception to vision and cognition, thus focussing interventions on visible elements and information that is processed through the eyes, such as labels, advertisements, and visual media (ibid.). Additionally, top-down policy geared towards information-giving has disempowering effects on consumers, resulting in information overload and consequent confusion (Longo et al., 2019). Accordingly, we invite policy and educational efforts to focus on all the senses, including touch, taste, and smell. We argue that all aspects of the body need to be engaged in the future research and policy so that the perception of consumption practices is broadened to encompass a holistic embodied view. This is particularly important in the case of children, who should be invited to experiment, thus stimulating children’s sensorial curiosity for healthy food. Unfortunately, much nutritional education in schools still focuses solely on passive learning of abstract, written materials (what is fat, protein, carbohydrates, etc.), eschewing sensorily engaging, experimental learning (Murimi et al., 2017). Experimentation and socialisation with heathy and sustainable foods within schools, and increasing familiarity with alternative food provisioning channels, is vital for overcoming the ontological security afforded by often familiar foods that constitute an imbalanced diet.

These reflections have also important managerial implications. Recognising the central role of the body in ethical consumption processes, those seeking to foster more sustainable and healthy diets should trial multi-dimensional initiatives that engage consumers from a multi-sensorial and embodied perspective. For instance, the matching of specific sounds with flavours modifies, and can enhance the multisensory tasting experience (Spence, 2017). Consequently, pairing sweet sounding music with tasting fruits and vegetables increases perceptions of the latter’s sweetness, as well as liking and probability of future consumption (Guedes et al., 2024). A multi-sensorial perspective thus appears promising for the design of more effective dietary change initiatives.

#### Avenue 4

A key element of practice theory concerns how multiple ways of performing compete, differ, and are routinized (Pekkanen, 2021). Practice theory recognises that practices are performed within broader constellations that become habitual and routinized (Moraes et al., 2017; Perera et al., 2018) and that constellations of practices can get misaligned, reconfigured, and destabilized (Phipps & Ozanne, 2017). The question then becomes: what embodied states are more conducive to destabilization, misalignment, and reconfiguration of constellations of practices? This question is central for policy debates, as transforming practices requires the creation of new bodily perceptions and memories: bodies need to be engaged in order to experiment with new practices (Wallenborn & Wilhite, 2014). Rather than designing interventions which take an individual, cognitive approach, there is a need for instigating experiences that reconcile conflicting embodied states, for example creating meals that are enjoyable sensory occasions for all family members, whilst also being healthy and sustainable.

## Data Availability

N/A.

## References

[CR1] Ajzen, I. (1991). The theory of planned behavior. *Organizational Behavior and Human Decision Processes,**50*(2), 179–211.

[CR2] Amilien, V., Discetti, R., Lecoeur, J. L., et al. (2022). European food quality schemes in everyday food consumption: An exploration of sayings and doings through pragmatic regimes of engagement. *Journal of Rural Studies,**95*, 336–349.

[CR3] Andorfer, V. A., & Liebe, U. (2012). Research on fair trade consumption—A review. *Journal of Business Ethics,**106*(4), 415–435.

[CR4] Bell, E., Winchester, N., & Wray-Bliss, E. (2021). Enchantment in business ethics research. *Journal of Business Ethics,**174*, 251–262.

[CR5] Böhm, S., Carrington, M., Cornelius, N., de Bruin, B., Greenwood, M., Hassan, L., & Shaw, D. (2022). Ethics at the centre of global and local challenges: Thoughts on the future of business ethics. *Journal of Business Ethics,**180*(3), 835–861. 10.1007/s10551-022-05239-2 PMC9533288 36212626

[CR6] Bourdieu, P. (1977). *Outline of a theory of practice*. Cambridge University Press.

[CR7] Bourdieu, P. (1990). *The logic of practice*. Stanford University Press.

[CR8] Braun, V., & Clarke, V. (2006). Using thematic analysis in psychology. *Qualitative Research in Psychology,**3*(2), 77–101.

[CR9] Bray, J., Johns, N., & Kilburn, D. (2011). An exploratory study into the factors impeding ethical consumption. *Journal of Business Ethics,**98*(4), 597–608.

[CR10] Bürgin, D., & Wilken, R. (2022). Increasing consumers’ purchase intentions toward fair-trade products through partitioned pricing. *Journal of Business Ethics,**181*(4), 1015–1040.

[CR11] Carey, L., Shaw, D., & Shiu, E. (2008). The impact of ethical concerns on family consumer decision-making. *International Journal of Consumer Studies,**32*(5), 553–560.

[CR12] Carrington, M., Chatzidakis, A., Goworek, H., & Shaw, D. (2021). Consumption ethics: A review and analysis of future directions for interdisciplinary research. *Journal of Business Ethics,**168*, 215–238.

[CR13] Carrington, M. J., Neville, B. A., & Whitwell, G. J. (2010). Why ethical consumers don’t walk their talk: Towards a framework for understanding the gap between the ethical purchase intentions and actual buying behaviour of ethically minded consumers. *Journal of Business Ethics,**97*(1), 139–158.

[CR14] Carrington, M. J., Neville, B. A., & Whitwell, G. J. (2014). Lost in translation: Exploring the ethical consumer intention–behavior gap. *Journal of Business Research,**67*(1), 2759–2767.

[CR15] Carrington, M. J., Zwick, D., & Neville, B. (2016). The ideology of the ethical consumption gap. *Marketing Theory,**16*(1), 21–38.

[CR16] Caruana, R., Carrington, M. J., & Chatzidakis, A. (2016). Beyond the attitude-behaviour gap: Novel perspectives in consumer ethics. *Journal of Business Ethics,**136*, 215–218.

[CR17] Chatzidakis, A., Hibbert, S., & Smith, A. P. (2007). Why people don’t take their concerns about fair trade to the supermarket: The role of neutralisation. *Journal of Business Ethics,**74*(1), 89–100.

[CR18] Coffin, J., & Egan-Wyer, C. (2022). The ethical consumption cap and mean market morality. *Marketing Theory,**22*(1), 105–123.

[CR19] Connolly, J., & Prothero, A. (2008). Green consumption: Life-politics, risk and contradictions. *Journal of Consumer Culture,**8*(1), 117–145.

[CR20] Corbin, J., & Strauss, A. (2014). *Basics of qualitative research: Techniques and procedures for developing grounded theory*. Sage Publications.

[CR21] d’Astous, A., & Legendre, A. (2009). Understanding consumers’ ethical justifications: A scale for appraising consumers’ reasons for not behaving ethically. *Journal of Business Ethics,**87*(2), 255–268.

[CR22] Dale, K., & Latham, Y. (2015). Ethics and entangled embodiment: Bodies–materialities–organization. *Organization,**22*(2), 166–182.

[CR23] ElHaffar, G., Durif, F., & Dubé, L. (2020). Towards closing the attitude-intention-behavior gap in green consumption: A narrative review of the literature and an overview of future research directions. *Journal of Cleaner Production,**122*, 556.

[CR24] EU Policy Lab. (2025). Unlocking the full potential of behavioural insights for policy. Available at https://publications.jrc.ec.europa.eu/repository/handle/JRC138028

[CR25] Evans, D. (2012). Beyond the throwaway society: Ordinary domestic practice and a sociological approach to household food waste. *Sociology,**46*(1), 41–56.

[CR26] Evans, D. (2019). What is consumption, where has it been going, and does it still matter? *The Sociological Review,**67*(3), 499–517.

[CR27] FAO, UNEP, WHO, and WOAH, (2022) Global Plan of Action on One Health. Towards a more comprehensive One Health, approach to global health threats at the human-animal-environment interface. Rome.

[CR28] Fischer, D., Reinermann, J. L., Mandujano, G. G., DesRoches, C. T., Diddi, S., & Vergragt, P. J. (2021). Sustainable consumption communication: A review of an emerging field of research. *Journal of Cleaner Production,**300*, Article 126880.

[CR29] Franco, C., & Ghisetti, C. (2022). What shapes the “value-action” gap? The role of time perception reconsidered. *Economia Politica,**39*(3), 1023–1053.

[CR30] Gamma, K., Mai, R., & Loock, M. (2020). The double-edged sword of ethical nudges: Does inducing hypocrisy help or hinder the adoption of pro-environmental behaviors? *Journal of Business Ethics,**161*(2), 351–373.

[CR31] Giddens, A. (1984). The constitution of society: Outline of the theory of structuration. Polity.

[CR32] Gordon, R., Harada, T., & Spotswood, F. (2022). The body politics of successful ageing in the nexus of health, well-being and energy consumption practices. *Social Science & Medicine,**294*, Article 114717. 35033799 10.1016/j.socscimed.2022.114717

[CR33] Govind, R., Singh, J. J., Garg, N., & D’Silva, S. (2019). Not walking the walk: How dual attitudes influence behavioral outcomes in ethical consumption. *Journal of Business Ethics,**155*, 1195–1214.

[CR34] Gruber, V., & Schlegelmilch, B. B. (2014). How techniques of neutralization legitimize norm-and attitude-inconsistent consumer behavior. *Journal of Business Ethics,**121*(1), 29–45.

[CR35] Guedes, D., Garrido, M. V., Lamy, E., & Prada, M. (2024). Disentangling cross-modality and affect in “sonic seasoning”: The effect of music associated with different degrees of sweetness and valence on food perception. *International Journal of Gastronomy and Food Science,**35*, Article 100879.

[CR36] Hassan, L. M., Shiu, E., & Shaw, D. (2016). Who says there is an intention–behaviour gap? Assessing the empirical evidence of an intention–behaviour gap in ethical consumption. *Journal of Business Ethics,**136*(2), 219–236.

[CR37] Heath, T., O’Malley, L., Heath, M., & Story, V. (2016). Caring and conflicted: Mothers’ ethical judgments about consumption. *Journal of Business Ethics,**136*(2), 237–250.

[CR38] Huff, A. D., & Cotte, J. (2016). The evolving family assemblage: How senior families “do” family. *European Journal of Marketing,**50*(5/6), 892–915.

[CR39] Jackson, P., Evans, D., Truninger, M., Baptista, J., & Nunes, N. C. (2022). Tasting as a social practice: A methodological experiment in making taste public. *Social & Cultural Geography,**23*(5), 739–756.

[CR40] Jacobsen, M. H., & Hansen, A. R. (2021). (Re) introducing embodied practical understanding to the sociology of sustainable consumption. *Journal of Consumer Culture,**21*(4), 747–763.

[CR41] Johnstone, M. L., & Tan, L. P. (2015). Exploring the gap between consumers’ green rhetoric and purchasing behaviour. *Journal of Business Ethics,**132*(2), 311–328.

[CR42] Longo, C., Shankar, A., & Nuttall, P. (2019). “It’s not easy living a sustainable lifestyle”: How greater knowledge leads to dilemmas, tensions and paralysis. *Journal of Business Ethics,**154*, 759–779.

[CR43] Mak, C. K., Lai, A. L., Tsaousi, C., & Davies, A. (2022). Mobilising the walking-with technique to explore mundane consumption practices: Practical and theoretical reflections. *Qualitative Market Research,**25*(3), 383–401.

[CR44] Mandalaki, E., & Fotaki, M. (2020). The bodies of the commons: Towards a relational embodied ethics of the commons. *Journal of Business Ethics,**166*(4), 745–760.

[CR45] Martens, L., Halkier, B., & Pink, S. (2014). Researching habits: Advances in linguistic and embodied research practice. *International Journal of Social Research Methodology,**17*(1), 1–9.

[CR46] Molander, S. (2017). Not just a mother: Embodied and positional aspects of consumer learning from a practice perspective. *Consumption Markets & Culture,**20*(2), 131–152.

[CR47] Molander, S., & Hartmann, B. J. (2018). Emotion and practice: Mothering, cooking, and teleoaffective episodes. *Marketing Theory,**18*(3), 371–390.

[CR48] Moraes, C., Carrigan, M., Bosangit, C., Ferreira, C., & McGrath, M. (2017). Understanding ethical luxury consumption through practice theories: A study of fine jewellery purchases. *Journal of Business Ethics,**145*(3), 525–543.

[CR49] Murimi, M. W., Kanyi, M., Mupfudze, T., Amin, M. R., Mbogori, T., & Aldubayan, K. (2017). Factors influencing efficacy of nutrition education interventions: A systematic review. *Journal of Nutrition Education and Behavior,**49*(2), 142–165. 27814976 10.1016/j.jneb.2016.09.003

[CR50] Neuman, N. (2019). On the engagement with social theory in food studies: Cultural symbols and social practices. *Food, Culture & Society,**22*(1), 78–94.

[CR51] Nicolini, D. (2012). *Practice theory, work, and organization*. Oxford University Press.

[CR52] Öberseder, M., Schlegelmilch, B. B., & Gruber, V. (2011). Why don’t consumers care about CSR?: A qualitative study exploring the role of CSR in consumption decisions. *Journal of Business Ethics,**104*(4), 449–460.

[CR53] O’Malley, L., & Prothero, A. (2006). Consuming families: Marketing, consumption and the role of families in the twenty-first century. *Journal of Marketing Management,**22*(9–10), 899–905.

[CR54] Papaoikonomou, E., Ryan, G., & Ginieis, M. (2011). Towards a holistic approach of the attitude behaviour gap in ethical consumer behaviours: Empirical evidence from Spain. *International Advances in Economic Research,**17*(1), 77–88.

[CR55] Pecoraro, M. G., & Uusitalo, O. (2014). Conflicting values of ethical consumption in diverse worlds–A cultural approach. *Journal of Consumer Culture,**14*(1), 45–65.

[CR56] Pekkanen, T. L. (2021). Institutions and agency in the sustainability of day-to-day consumption practices: An institutional ethnographic study. *Journal of Business Ethics,**168*(2), 241–260.

[CR57] Perera, C., Auger, P., & Klein, J. (2018). Green consumption practices among young environmentalists: A practice theory perspective. *Journal of Business Ethics,**152*(3), 843–864.

[CR58] Phipps, M., & Ozanne, J. L. (2017). Routines disrupted: Reestablishing security through practice alignment. *Journal of Consumer Research,**44*(2), 361–380.

[CR59] Pink, S. (2009). *Doing Sensory Ethnography*. Sage.

[CR60] Reckwitz, A. (2002). Toward a theory of social practices: A development in culturalist theorizing. *European Journal of Social Theory,**5*(2), 243–263.

[CR61] Sahelices-Pinto, C., Lanero-Carrizo, A., & Vázquez-Burguete, J. L. (2021). Self-determination, clean conscience, or social pressure? Underlying motivations for organic food consumption among young millennials. *Journal of Consumer Behaviour,**20*(2), 449–459.

[CR62] Schatzki, T. (2001). *The practice turn in contemporary theory*. Routledge.

[CR63] Shaw, D., McMaster, R., Longo, C., & Özçaglar-Toulouse, N. (2017). Ethical qualities in consumption: Towards a theory of care. *Marketing Theory,**17*(4), 415–433.

[CR64] Shaw, D., McMaster, R., & Newholm, T. (2016). Care and commitment in ethical consumption: An exploration of the ‘attitude–behaviour gap.’ *Journal of Business Ethics,**136*(2), 251–265.

[CR65] Southerton, D. (2013). Habits, routines and temporalities of consumption: From individual behaviours to the reproduction of everyday practices. *Time & Society,**22*(3), 335–355.

[CR66] Spence, C. (2017). *Gastrophysics: The New Science of Eating*. Penguin Books.

[CR67] Spotswood, F., Steele, J., Androulakis-Korakakis, P., & Lucas, A. (2024). The dynamics of teleoaffective configuration in practice adaptation. *Marketing Theory,**24*(3), 499–524.

[CR68] Subramani, S. (2022). Moral habitus: An approach to understanding embedded disrespectful practices. *Developing World Bioethics,**22*(2), 94–104. 33258204 10.1111/dewb.12301

[CR69] Szmigin, I., Carrigan, M., & McEachern, M. G. (2009). The conscious consumer: Taking a flexible approach to ethical behaviour. *International Journal of Consumer Studies,**33*(2), 224–231.

[CR71] Trees, R., & Dean, D. M. (2018). Physical and emotional nourishment: Food as the embodied component of loving care of elderly family relatives. *European Journal of Marketing,**52*(12), 2405–2422.

[CR72] Wallenborn, G., & Wilhite, H. (2014). Rethinking embodied knowledge and household consumption. *Energy Research & Social Science,**1*, 56–64.

[CR73] Wallendorf, M., & Arnould, E. J. (1991). “We gather together”: Consumption rituals of thanksgiving day. *Journal of Consumer Research,**18*(1), 13–31.

[CR74] Warde, A. (2005). Consumption and theories of practice. *Journal of Consumer Culture,**5*(2), 131–153.

[CR75] Warde, A. (2014). After taste: Culture, consumption and theories of practice. *Journal of Consumer Culture,**14*(3), 279–303.

[CR76] Weckroth, K., & Närvänen, E. (2024). Activity focus groups–a discursive, practical and social method for studying consumption practices. *Qualitative Market Research,**27*(2), 212–230.

[CR77] Welch, D., & Warde, A. (2015). Theories of Practice and Sustainable Consumption. In L. Reisch & J. Thøgersen (Eds.), *Handbook of research on sustainable consumption* (pp. 84–100). Edward Elgar Publishing.

[CR78] Yan, L., Keh, H. T., & Wang, X. (2021). Powering sustainable consumption: The roles of green consumption values and power distance belief. *Journal of Business Ethics,**169*, 499–516.

[CR79] Zollo, L. (2021). The consumers’ emotional dog learns to persuade its rational tail: Toward a social intuitionist framework of ethical consumption. *Journal of Business Ethics,**168*(2), 295–313.

